# Emphysematous pyelonephritis with perinephric extension and fistulous communication to the descending colon: a rare CT imaging diagnosis

**DOI:** 10.1093/bjrcr/uaag022

**Published:** 2026-05-21

**Authors:** Shweta Gaikwad, Eshan Thotwe

**Affiliations:** Department of Radiology, Jehangir Hospital, Pune, Maharashtra 411001, India; Department of Radiology, Jehangir Hospital, Pune, Maharashtra 411001, India

**Keywords:** emphysematous pyelonephritis, reno-colic fistula, CT, perinephric abscess, gas-forming renal infection

## Abstract

Emphysematous pyelonephritis (EPN) is a severe necrotizing infection characterized by gas formation within the renal parenchyma and surrounding tissues. Although perinephric extension is well recognized, fistulous communication with the adjacent bowel is exceedingly rare. We report a case of a 58-year-old woman presenting with fever, left flank pain, uncontrolled diabetes mellitus, and acute kidney injury. Initial non-contrast CT KUB showed left EPN with a large perinephric air-containing collection and loss of fat planes with the adjacent descending colon, raising suspicion of a reno-colic fistula. Following DJ stenting, a repeat CT scan with administration of rectal contrast demonstrated direct contrast leakage into the perinephric collection, confirming fistulous communication. Subsequent colonoscopy corroborated the imaging findings. This case highlights the importance of sequential CT imaging and the role of rectal contrast in confirming rare fistulous complications of EPN.

## Introduction

Emphysematous pyelonephritis (EPN) is a rare, life-threatening necrotizing renal infection characterized by gas formation within the renal parenchyma and surrounding tissues.^1^ It most commonly affects patients with poorly controlled diabetes mellitus and carries significant morbidity and mortality.^1^ The historical foundation of gas-forming renal infections dates back to 1898, when Kelly and MacCallum reported the first case of pneumaturia.^2^ Patients with urinary obstruction, diabetes, or immunocompromised status are more likely to develop complicated infections and abscess.^3^ The initial clinical manifestation of these patients may be insidious, but rapid progression to sepsis can occur in the absence of early medical intervention.^4^

Imaging in the early stage of the symptoms is important in making the diagnosis of acute renal infections and helps identify complications such as abscess formation.^5^

Traditional modalities like intravenous urogram and ultrasound have been used in the assessment of patients with suspicion of renal infections, allowing detection of calculi, obstruction, and incomplete bladder emptying. These imaging techniques, however, have limitations in the evaluation of renal inflammation and infection in the adult.^6^

A CT scan is the gold standard imaging modality for diagnosis and classification, allowing for the accurate assessment of disease extent and complications.^7^ According to the Huang and Tseng classification, extension into the perinephric space represents advanced disease (Class 3A), while extension into the pararenal space or adjacent tissues is categorized as Class 3B.^2^

Fistulous communication between renal or perinephric infections and adjacent bowel is extremely uncommon and is more frequently associated with chronic inflammatory conditions such as xanthogranulomatous pyelonephritis.^8^ Historically, as noted by Miller,^9^ no instance of this condition has been reported as a result of a primary bowel lesion; instead, these fistulas are incident to a chronic suppurative process of the kidney with associated peri-nephritis or perinephric abscess formation. The mechanism typically involves progressive inflammation, abscess formation, and erosion into adjacent bowel loops.^9^

We present a rare case of EPN complicated by perinephric extension and secondary reno-colic fistula, with emphasis on the role of tailored CT imaging in diagnosis.

## Case presentation and imaging findings

A 58-year-old woman presented with fever, vomiting, loose stools, and left flank pain. She had a known history of poorly controlled diabetes mellitus and deranged renal function.

Laboratory evaluation revealed leukocytosis with a total leukocyte count of 16 000 cells/mm³ and neutrophilic predominance along with elevated inflammatory markers, including C-reactive protein (CRP), erythrocyte sedimentation rate (ESR), and procalcitonin (PCT). The patient had severe acute kidney injury with a serum creatinine of 8.9 mg/dL on admission. She was managed with sustained low-efficiency daily dialysis (SLEDD), following which creatinine temporarily decreased to 0.5 mg/dL but subsequently rose again to 4.0 mg/dL over the next 5 days.

Urine analysis demonstrated marked pyuria, leukocyte esterase positivity (3+), nitrite positivity, significant bacteriuria, mild proteinuria, and microscopic hematuria.

## Imaging findings

**Figure 1:**
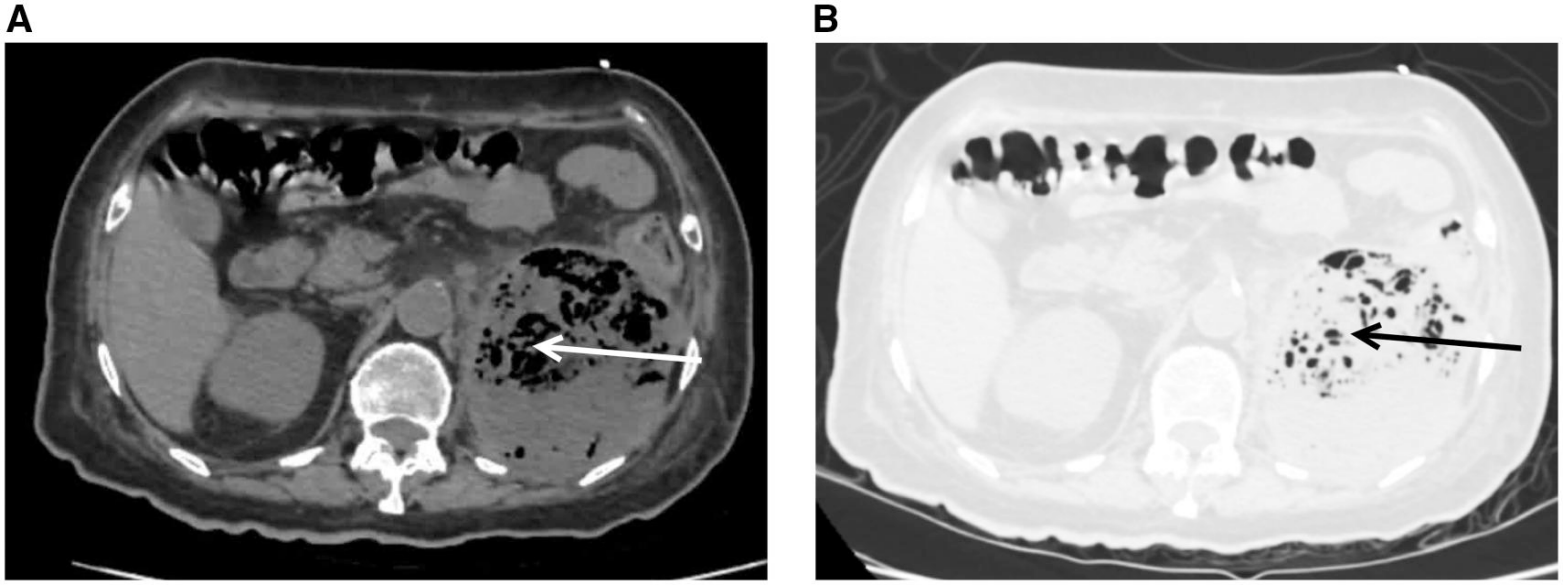
Axial non-contrast CT images (A-Soft window) and (B-Lung window) demonstrating extensive gas within the left renal parenchyma (arrows) consistent with emphysematous pyelonephritis.

**Figure 2:**
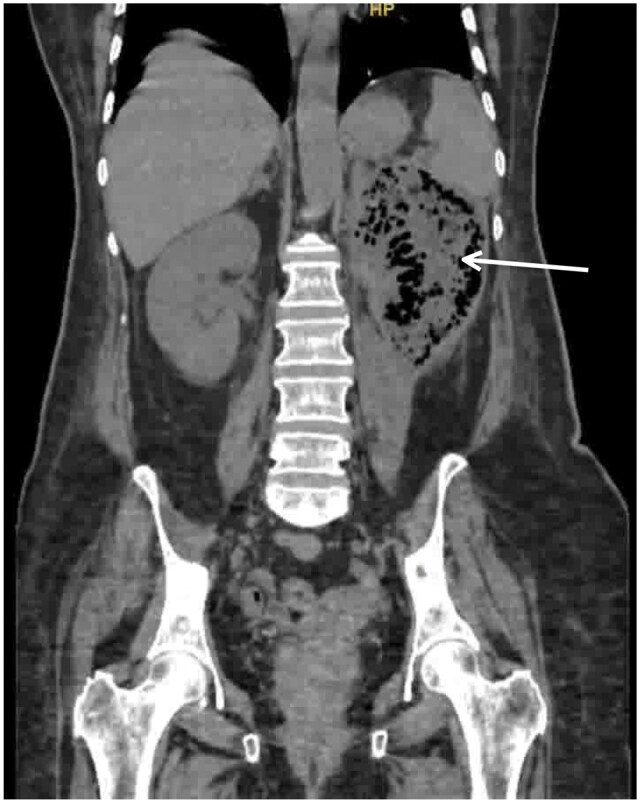
Coronal non-contrast CT image showing air-containing collection involving the left kidney with extension into the perinephric space (arrow).

Non-contrast CT KUB was performed due to significantly deranged renal function (serum creatinine 8.9 mg/dL) and demonstrated extensive gas within the left renal parenchyma involving the upper and mid poles with perinephric extension, consistent with EPN (Figures 1 and 2).

**Figure 3:**
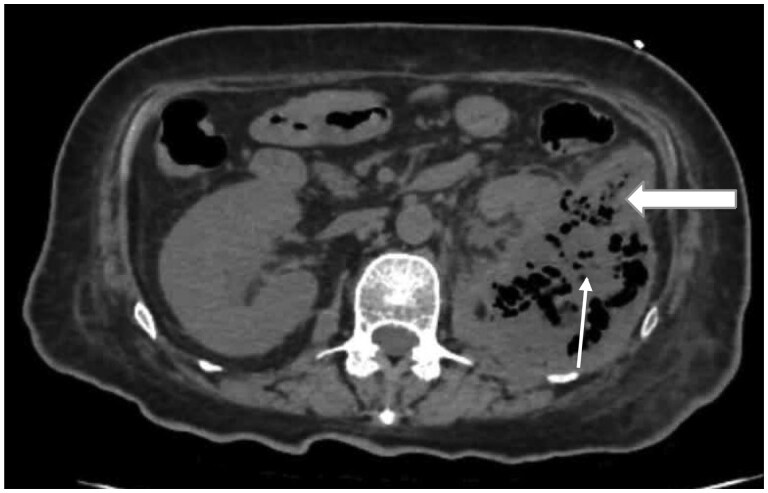
Axial non-contrast CT image demonstrating perinephric air-containing collection (thin arrow) abutting the descending colon with focal mural irregularity (Block arrow) raising suspicion of fistulous communication.

At the level of the proximal descending colon, focal mural irregularity with loss of fat planes between the colon and perinephric collection was noted, raising suspicion of fistulous communication (Figure 3).

**Figure 4:**
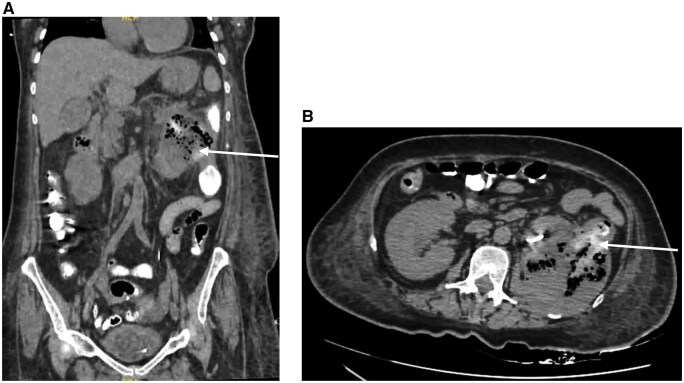
Coronal (A) and axial (B) CT images following rectal contrast administration demonstrating leakage of contrast (arrows) from the descending colon into the perinephric collection, confirming fistulous communication. Focal mural defect in the posteromedial wall of the descending colon (arrow in image B) corresponding to the site of fistulous communication.

Following DJ ureteral stenting, CT with rectal contrast demonstrated contrast leakage into the perinephric collection with a focal defect in the posteromedial wall of the descending colon, confirming reno-colic fistula (Figure 4).

**Figure 5:**
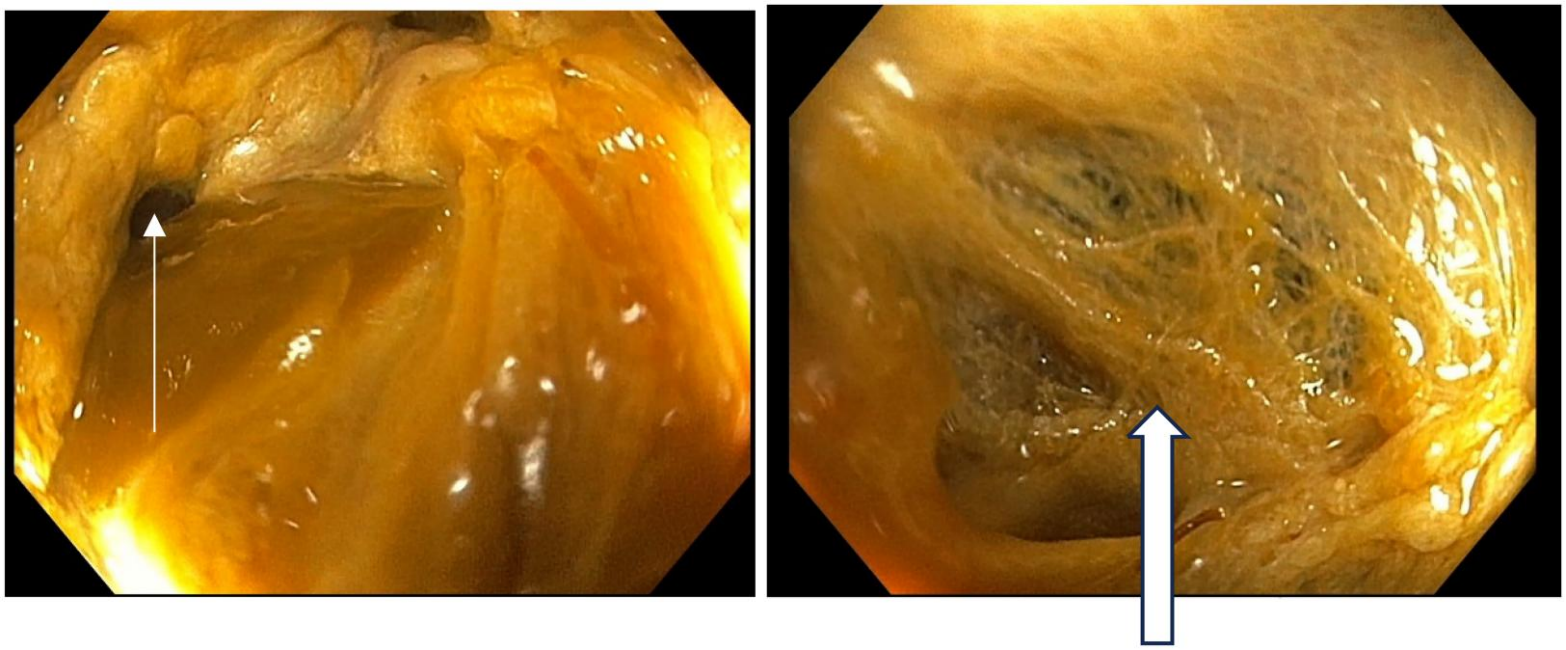
Colonoscopy images showing fistulous opening at proximal descending colon (White arrow) and large area of fibrous gauge-like tissue around fistulous opening (Block arrow).

Colonoscopy demonstrated a fistulous opening approximately 40 cm from the anal verge with surrounding fibrotic slough-covered tissue, correlating with imaging findings (Figure 5).

## Treatment and outcome

The patient was managed with urinary decompression via DJ stenting, renal support with dialysis, and intensive medical therapy including intravenous antibiotics, insulin, and supportive care.

Although there was initial clinical improvement, the patient subsequently developed worsening sepsis and recurrent renal impairment. Microbiological evaluation of the pus sample acquired during colonoscopy demonstrated extensively drug-resistant *Klebsiella pneumoniae.* Surgical intervention was advised but was declined. Despite intensive care and supportive treatment, the prognosis remained poor, and the family opted for end-of-life care, following which the patient was discharged against medical advice.

## Discussion

EPN is a severe necrotizing infection caused by gas-forming organisms such as *Escherichia coli* and *K pneumoniae*.^1^ CT imaging plays a central role in diagnosis and allows accurate assessment of disease extent and complications.^7^

Renal parenchymal involvement of more than 50% on initial imaging is a significant and independent predictor of poor renal function.^10^

Radiologically, this case can be classified using 2 established systems. According to Huang and Tseng, extension of gas into adjacent structures, including the descending colon, corresponds to Class 3B disease, which is associated with more severe clinical outcomes.^2^ As per Wan et al., the presence of gas associated with a fluid collection is consistent with Type II EPN.^1^

Fistula formation in renal infections is rare and most commonly associated with chronic inflammatory conditions such as xanthogranulomatous pyelonephritis.^8^ The underlying mechanism involves prolonged inflammation, perinephric abscess formation, and eventual erosion into adjacent bowel structures.^5^ Other reported etiologies of reno-colic fistula include post-interventional states such as nephrolithotomy and renal malignancies such as renal cell carcinoma,^11^^,^^12^

In this case, fistula formation occurred secondary to perinephric extension of acute emphysematous infection rather than chronic renal disease.

A key highlight is the stepwise radiological approach where an initial CT raised suspicion, while a rectal contrast-administered CT confirmed the diagnosis through demonstration of contrast leakage. Colonoscopy provided direct visualization of the fistulous tract. This underscores the value of tailored imaging protocols, particularly the use of rectal contrast in suspected enteric fistulae.

## Learning points

- Emphysematous pyelonephritis is a life-threatening infection requiring early diagnosis.
- Perinephric extension indicates advanced disease and poor prognosis.
- Reno-colic fistula is a rare complication of EPN.
- Loss of fat planes and bowel wall irregularity on CT scan should raise suspicion of fistula.
- Rectal contrast-enhanced CT scan is valuable in confirming fistulous communication.
